# Word of Mouth and Online Reviews Are More Influential Than Social Media for Patients When Selecting a Sports Medicine Physician

**DOI:** 10.1016/j.asmr.2022.04.022

**Published:** 2022-05-30

**Authors:** Dylan N. Greif, Harsh A. Shah, Dylan Luxenburg, Blake H. Hodgens, Anabel L. Epstein, Lee D. Kaplan, Julianne Munoz, Michael Letter, Michael G. Baraga

**Affiliations:** a Department of Orthopaedics, University of Rochester, Rochester, New York, U.S.A; b Department of Orthopaedic Surgery, University of Miami Hospital, Miami, Florida, U.S.A; c Leonard M. Miller School of Medicine, University of Miami, Miami, Florida, U.S.A; d University of Miami Sports Medicine Institute, Coral Gables, Florida, U.S.A

## Abstract

**Purpose:**

To (1) identify the percentage of patients seen in an orthopaedic sports medicine practice who use social media and (2) identify the role that social media has in physician selection as compared with other factors.

**Methods:**

After institutional review board approval was received, new patients aged 18 years or older who attended a single orthopaedic sports medicine office from February 2020 to May 2021 were identified for inclusion. Sociodemographic information was recorded, and each patient was asked to fill out a questionnaire that assessed social media usage and online resources used to choose and formulate opinions regarding the patient’s provider.

**Results:**

Two hundred patients met the inclusion criteria and completed the questionnaire. Of these, 96.5% reported social media use. The most common online method of searching for and identifying a physician was Google (50.5%). Social media outlets such as Facebook, Instagram, or LinkedIn were only used 15.5% of the time to search for and select a physician. Older patients were more likely to use recommendations from friends and family in their consideration when selecting a physician.

**Conclusions:**

Despite almost all participants stating that they use social media, only 15.5% of patients reported that they used social media to search for and potentially select their physician. Our study suggests that although social media can be a helpful tool for patient education, other factors such as physician education and physician reputation through word-of-mouth referrals, online reviews, and online ratings seem to play a larger role in the patient’s selection of his or her physician.

**Clinical Relevance:**

This information may be of value to orthopaedic surgeons looking for ways to build their patient base, online reputation, or other aspects of their practice on the Internet.

Physicians have an opportunity by using social media and the Internet to promote their services and qualifications to attract potential patients, provide educational content that otherwise may have required an office visit, and improve their reputation through physician online rating systems.^1^, ^2^, ^3^, ^4^, ^5^ The influence of online resources on medicine is highlighted by the fact that approximately 80% of patients will use the Internet to obtain health information at some point in their lifetimes.^6^ Despite 63% of patients reporting that they trust their physician the most for obtaining health information, almost half of patients reported that they use the Internet to research their tentative diagnosis prior to seeing a physician.^7^

Traditional methods of patient recruitment have focused on establishing primary care referrals and word-of-mouth recommendations; however, as competition for generating and maintaining a patient population increases, a social media presence affords providers a free and easily accessible resource to increase patient engagement.^8^ A recent representative sample from across the United States identified at least 1 online profile for 94.3% of orthopaedic surgeons.^9^ Despite recent studies showing that board certification, recognition for a specific area of expertise, availability of onsite imaging services, and in-network provider status all have a profound influence on orthopaedic surgeon selection by patients,^10^^,^^11^ the effect that a social media or online presence has on how patients choose, refer, and rate their orthopaedic surgeons is still unknown.

The purpose of our study was to (1) identify the percentage of patients seen in an orthopaedic sports medicine practice who use social media and (2) identify the role that social media has in physician selection as compared with other factors. We hypothesized that social media would influence at least half of patients in the process of physician selection.

## Methods

### Study Population

After institutional review board approval was received, new patients who attended a single orthopaedic sports medicine clinic from February 2020 to May 2021 were approached by a medical student on the research team (D.L., B.H., and A.E.) at the conclusion of their visit. Patients younger than 18 years were excluded because, as they are minors, special consent would be required from a guardian. Verbal consent was obtained from each included patient after a detailed explanation of the study objectives and protocol, and patients were asked to fill out a paper-based questionnaire that included basic sociodemographic identifiers and assessed what information and/or sources patients use to choose and formulate opinions regarding their provider. Patients who agreed to participate were included regardless of the reason for their visit, and participation was voluntary. Participants did not receive any incentive, financial or otherwise. Prior to enrollment, it was confirmed that all 3 attending orthopaedic surgeons (J.M., L.D.K., M.G.B.) had readily accessible social media profiles and were listed online on our university website. There were no specific patient recruitment tactics, and no outside agencies were involved in maintaining the physicians’ social media accounts.

### Questionnaire

**Fig 1:**
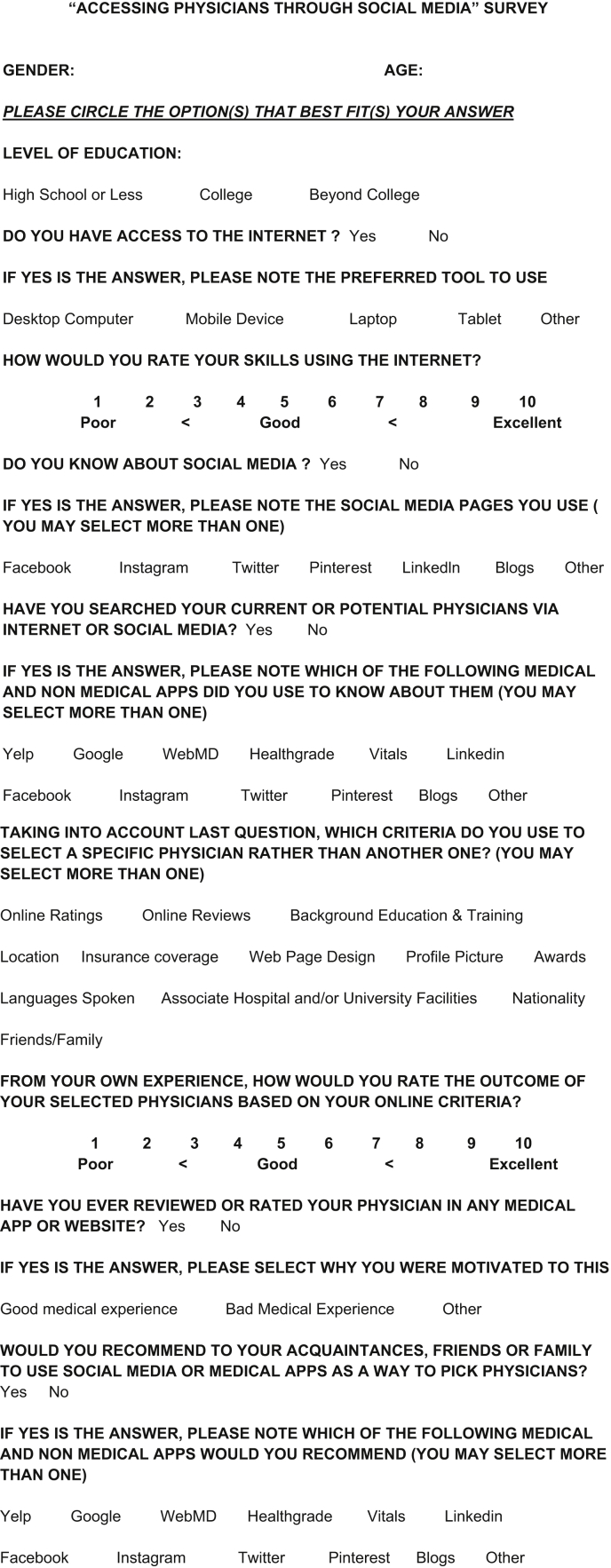
Survey provided to patients after clinic visit.

Patients were encouraged to answer all questions as truthfully as possible and were given the option to leave questions unanswered if they did not apply (Fig 1). Data collected included sociodemographic identifiers (age, sex, and educational status) in addition to variables related to patients’ access to the Internet, preferred Internet usage tool, and self-reported skills using the Internet. Answer choices were recorded as either continuous variables on a scale from 1 to 10, with 10 being the highest rating, or as categorical variables.

The questionnaire asked patients to specify which social media pages they currently use, including Facebook, Instagram, Twitter, Pinterest, LinkedIn, blogs, or other. Patients then identified whether they have ever searched for a current or potential physician via the Internet or social media. If patients specified that they have run a search, they then selected which medical and nonmedical applications they used, including Yelp, Google (Alphabet), WebMD, Healthgrades, Vitals, LinkedIn, Facebook, Instagram, Twitter, Pinterest, blogs, or other.

Next, the questionnaire asked the patients to identify the criteria they use to select their physician and to rate the outcome of their medical experience based on their identified criteria. The criteria available for patient selection were as follows: online ratings, online reviews, background education and training, location, insurance coverage, web page design, profile picture, awards, languages spoken, associate hospital and/or university facilities, nationality, or friends/family. Patients identified whether they have ever reviewed or rated their physician on a medical application or website and, if so, whether they were motivated to do so based on a good or bad medical experience. Finally, patients identified whether they would endorse social media or medical applications to select a potential physician and, if so, which applications they would endorse.

### Statistical Analysis

Statistical analysis of all data was performed using IBM SPSS Statistics software (version 26). Data were analyzed using binary logistic regression models. *P* < .05 was considered statistically significant.

## Results

### Sociodemographic and Clinical Characteristics

Two hundred patients verbally consented and completed the questionnaire; 64.5% (n = 129) were men and 35.5% (n = 71) were women (Table 1). The mean age of the participants was 40.7 ± 15.7 years (range, 18-87 years). Regarding education, 16% of participants (n = 32) completed high school or a lower level of education, 45% (n = 90) completed college, and 39% (n = 78) completed a level of education beyond college. Of the participants, 98.5% had access to the Internet; their self-reported rating of their skill using the Internet was, on average, 8.5 of 10 (Table 2). Among participants who reported having Internet access, the most common method of accessing the Internet was through a mobile device (64.5%), followed by laptop use (42.5%) and desktop computer use (19.5%).

**Table 1:** Participants’ Demographic Characteristics

Characteristic	n	%
Sex		
Male	129	64.5
Female	71	35.5
Level of education		
High school or less	32	16
College	90	45
Beyond college	78	39
Age		
18-35 yr	84	42
36-55 yr	71	35.5
≥56 yr	45	22.5

**Table 2:** Participants’ Survey Answer Choices

Survey Questions and Answer Options	n	%	Average Reported Score (of 10)
Participants who have access to Internet	197	98.5	
Participants’ self-reported skills for using Internet			8.5
Preferred Internet usage tool (participants can select >1 choice)			
Desktop computer	39	19.5	
Mobile device	129	64.5	
Laptop	85	42.5	
Tablet	19	9.5	
Other	2	1	
Participants who know about social media	193	96.5	
Social media pages participants use (participants can select >1 choice)			
Facebook	116	58	
Instagram	141	70.5	
Twitter	61	30.5	
Pinterest	25	12.5	
LinkedIn	62	31	
Blogs	10	5	
Other	28	14	
Participants who have searched for current or potential physicians via Internet or social media	123	61.5	
Medical and nonmedical applications participants use to search for current or potential physicians via Internet or social media (participants can select >1 choice)			
Yelp	8	4	
Google	101	50.5	
WebMD	36	18	
Healthgrades	27	13.5	
Vitals	8	4	
LinkedIn	12	6	
Facebook	9	4.5	
Instagram	10	5	
Twitter	0	0	
Pinterest	2	1	
Blogs	3	1.5	
Other	15	7.5	
Participants’ criteria used to select specific physician (participants can select >1 choice)			
Online ratings	61	30.5	
Online reviews	84	42	
Background education and training	103	51.5	
Location	42	21	
Insurance coverage	62	31	
Web page design	6	3	
Profile picture	4	2	
Awards	8	4	
Languages spoken	8	4	
Associate hospital and/or university facilities	49	24.5	
Nationality	1	0.5	
Friends/family	78	39	
Participants’ self-reported outcome of selected physicians based on online criteria			8.4
Participants who have reviewed or rated their physician via any medical application or website	39	19.5	
Participants’ motivation to review or rate their physician			
Good medical experience	37	18.5	
Bad medical experience	2	1	
Other	4	2	
Participants who have recommended acquaintances, friends, or family to use social media or medical applications as method to select physicians	142	71	
Medical and nonmedical applications participants recommend to acquaintances, friends, or family to use as method to select physicians (participants can select >1 choice)			
Yelp	16	8	
Google	114	57	
WebMD	60	30	
Healthgrades	31	15.5	
Vitals	10	5	
LinkedIn	22	11	
Facebook	18	9	
Instagram	26	13	
Twitter	6	3	
Pinterest	1	0.5	
Blogs	4	2	
Other	14	7	

### Social Media Usage

Almost all participants were aware of social media and reported that they use social media (96.5%). The most common social media platform used was Instagram (70.5%), followed by Facebook (58%) and LinkedIn (31%) (Table 2). Regression analysis showed that increased age led to decreased knowledge about social media and decreased use of certain social media outlets such as Instagram (*P* < .001) and Twitter (*P* < .001). When stratified by age, participants’ social media usage varied extensively, as outlined in Table 3.

**Table 3:** Frequency of Social Media and Website Usage, Searches, and Recommendations by Age Group

	Age 18-35 yr	Age 36-55 yr	Age ≥ 56 yr
Frequency of social media usage by modality, %			
Facebook	57	62	53
Instagram	86	72	40
Twitter	40	30	13
LinkedIn	27	38	27
Frequency of searching for physician by website, %			
Google	56	49	42
WebMD	18	24	9
Healthgrades	7	14	24
Frequency of recommending physician by website, %			
Google	65	56	42
WebMD	31	35	20
Healthgrades	12	23	11

### Web Usage and Selection Criteria

Of all participants, 61.5% reported that they have searched for their physician on social media or the Internet, with the most common method being Google (50.5%), followed by WebMD (18%) and Healthgrades (13.5%). Patients used social media platforms such as Facebook, Instagram, or LinkedIn only 15.5% of the time to search for and choose their physician. Stratification of the utilization of websites to search for a potential physician by participant age is outlined in Table 3. Factors outside of both the Internet and social media were important to patients, including physician background, education, and training (51.5%); online reviews (42%); referrals from family or friends (39%); online ratings (30.5%); insurance coverage (31%); and associated hospital or university affiliation (24.5%). Patients rated the quality of the outcome of their physician encounter as 8.4 of 10, on average, based on their preferred criteria for physician selection. Older patients were less likely to use online reviews (*P* = .01) and online ratings (*P* = .019) whereas they were more likely to use recommendations from friends and family in their consideration (*P* = .024).

### Factors Considered When Rating or Recommending Physicians

Only 19.5% of participants reported that they had previously rated their physician online, with almost 95% of these patients motivated to rate their physician based on a good clinical experience. Increased age also led to increased odds of rating a physician online (*P* = .017), especially if the medical experience was a positive one (*P* = .016).

Finally, 71% of patients would recommend that their friends and family use social media or Internet sources to select a physician, with most recommending Google (57%), followed by WebMD (30%) and Healthgrades (15.5%). Age-specific stratification of recommended resources is outlined in Table 3. Men were almost 200% more likely to recommend social media or Internet sources to select a physician when compared with women (*P* = .01). Increased age led to a decrease in the odds of recommending Google (*P* = .006) and Twitter (*P* = .04).

## Discussion

Almost two-thirds of the patients in our study (61.5%) reported having searched for their physician via the Internet or social media, consistent with previous literature showing increased Internet reliance among patients.^6^^,^^7^^,^^11^ However, despite nearly all of the surveyed orthopaedic sports medicine patients (96.5%) currently having access to and using social media, only 15.5% reported using social media platforms such as Instagram, Twitter, and Facebook as a tool to select their physician. Rather, among those who reported searching for their physician, Internet-based websites including Google and WebMD were used much more often (Table 2). Google is a widely used platform that is known as a way to research and find answers to common questions whereas social media is less known for this reason. An interesting finding in our study was that whereas younger patients were more likely to rely on online ratings and reviews to find physicians, older patients relied more on friends and family in their consideration. Despite this, older patients were more likely to rate their physician online, especially if they had a positive medical experience.

The social media platforms most commonly used by patients included Instagram (70.5%), Facebook (58%), and LinkedIn (31%); however, few patients used these platforms specifically to search for their physicians (Instagram, 5%; Facebook, 4.5%; and LinkedIn, 6%) (Table 2). When data were stratified by age group, Instagram and Twitter were primarily used by the younger age groups whereas Facebook was used by over 50% of participants in each age group (Table 3). More young individuals are choosing to utilize social media, thus providing an age group for orthopaedic sports medicine physicians to target with promotional advertisements and educational content.

When it came to choosing a physician, our study participants did not prioritize their provider’s social media presence. Instead, they prioritized physician reputation through reviews and ratings, as well as word-of-mouth recommendations from friends and family in choosing their physician. This prioritization of family and friend recommendations was even more pronounced among older individuals. This finding is consistent with previous research that suggested a similar trend: In a study of 1,077 orthopaedic patients selecting a sports medicine physician, radio, television, and Internet advertisements were ranked the lowest in importance out of 19 factors.^11^ Despite physicians’ internet and social media presence not being the most important factor to patients, most patients (71%) indicated that they would recommend that their friends and family use these resources to select a physician, suggesting the value in curating a representative online presence.

Taking these findings into consideration, physicians should consider prioritizing patient experience, rather than their social media presence, for patient recruitment, given that the most effective means is based on the experiences of previous patients. Despite only a small percentage of participants reporting that they had previously rated their physician online, almost all of these patients (95%) were motivated to rate their physician based on a good clinical experience, further supporting the idea of prioritizing patient experience and facilitating online reviews.

A social media presence certainly has an important role in orthopaedic surgery aside from recruiting patients^4^^,^^12^^,^^13^ because there is value to curating both the professional and social presences to best align with patient preferences and notion of use. A recent study by Sama et al.^14^ investigated the impact of social media on orthopaedic sports medicine surgeons, finding that 62.4% of the included surgeons had at least one form of social media and that social media usage by the surgeon correlated with a higher overall online rating by patients. Although these findings may draw appeal to surgeons to begin utilizing social media accounts, our study suggests that only a small percentage of patients are using social media to choose their physicians (15.5%).

In a recent survey of orthopaedic patients performed by Curry et al.,^4^ 51% of patients reported using social media to gain insight into their medical condition, with higher use among younger patients. This finding provides physicians an opportunity to use social media platforms for patient education and dissemination of accurate information. This is consistent with a previous review performed by Rolls et al.^12^ suggesting that health care providers view social media platforms as portals for disseminating knowledge and citing clinically relevant and quality information. Access to accurate information is of utmost importance because most patients use the Internet to obtain health information at some point in their lifetimes.^6^ These findings parallel our study findings that social media is not a high-yield means of patient recruitment but rather should be used for connecting with patients, providing education, and displaying information about a physician’s practice and areas of expertise.

Outside of the aforementioned uses of social media, many orthopaedic social media influencers have begun to share videos of operative techniques, as well as discussions of imaging, cases, and the latest literature.^13^ Of note, preoperative education of orthopaedic patients is associated with increased patient satisfaction, improved quality of life, enhanced continuity of care, fewer complications, maximized independence and empowerment, better adherence to the plan of care, and decreased anxiety.^15^ These findings further highlight the significant benefit that social media can provide both orthopaedic providers and their patients.

As with any form of physician advertisement and patient recruitment strategy, there are inherent risks associated with social media use, including violations of patient privacy. Physicians must be mindful while using social media to adhere to patient privacy laws and maintain a high regard for medical-legal ethics.^8^ Additionally, with a growing social media presence, physicians may find it difficult to manage their own accounts. The consequences of inappropriate or unprofessional behavior can be swift and severe; therefore, clinical physicians and surgeons alike must work hard to understand the implications of social media use to avoid negative repercussions and maintain professionalism. Tenets for doing so include understanding institutional and professional social media policies, knowing one’s audience, policing one’s profile, continuing to learn, and always being cognizant of one’s digital footprint.^16^ In doing so, physicians can utilize social media as a fruitful educational and connection tool, rather than a reflection of unprofessionalism.

### Limitations

One inherent limitation of our study is that all of the questionnaire participants were seen at the same academic center in South Florida. Therefore, our study population may represent a single homogeneous group of patients, which limits the generalizability of our conclusions to patients who live in and visit medical centers or practice types in different regions or states. A second limitation of this study comes from patients not being differentiated based on health maintenance organization, preferred provider organization/Medicare, or self-pay status. Health maintenance organization insurance plans typically cover specific providers within a predetermined health care network, whereas preferred provider organization insurance plans typically provide patients with greater flexibility in selecting their health care providers. Consequently, some patients in our study may have been limited in selecting their health care providers based on the patients’ insurance coverage or lack thereof. Regarding the generalizability of this study, it is important to note that in countries that utilize a public health care system, referrals often predominate as the primary mode for selection of a specialist. Additionally, the patients who voluntarily participated in the survey may have had different perspectives than those who declined to participate. This represents potential bias among those who completed the survey. The exclusion of patients younger than 18 years represents further bias because these patients may have used social media differently than the included cohort. Finally, although all 3 orthopaedic surgeons included in the study maintained an online presence and social media profiles, the degree of online engagement may vary and, thus, may not be representative of all levels of social media usage and engagement.

## Conclusions

Despite almost all participants stating that they use social media, only 15.5% of patients reported that they used social media to search for and potentially select their physician. Our study suggests that although social media can be a helpful tool for patient education, other factors such as physician education and physician reputation through word-of-mouth referrals, online reviews, and online ratings seem to play a larger role in the patient’s selection of his or her physician.

## Supplementary Data

ICMJE author disclosure forms
